# THAT’S ANTHROPOLOGY: CONFRONTING AGEISM AND STUDENT DISCOMFORT IN SERVICE LEARNING

**DOI:** 10.1093/geroni/igae098.3897

**Published:** 2024-12-31

**Authors:** S M Cho, Cara Williams, Andrea Freidus, Dena Shenk

**Affiliations:** University of North Carolina at Charlotte, Charlotte, North Carolina, United States; University of North Carolina at Charlotte, Charlotte, North Carolina, United States; Michigan State University, Michigan State University, Michigan, United States; University of North Carolina at Charlotte, Charlotte, North Carolina, United States

## Abstract

Undergraduate anthropology majors participated in a 15-hour, semester-long service-learning project at an adult day and health care center during Spring 2024. While a research-based model of intergenerational service-learning has been incorporated in the undergraduate gerontology program curriculum at UNC Charlotte for 25 years, this was the first time anthropology undergraduates engaged in intergenerational research as part of their senior seminar. With no prior coursework in gerontology, and limited intergenerational experiences, anthropology senior seminar students began the semester with uncertainty and ageist biases. The founding director of the gerontology program, a professor emerita of anthropology, guided students’ practical development by providing lectures, leading exercises, and facilitating discussions throughout the semester. During the course, the students learned about and confronted complex presentations of ageism. Their time at the day center involved socializing with participants, assisting staff, creating and tending a therapeutic garden, working with participants to create memory boxes, and building rapport. These steps were essential for building a reciprocal, collaborative intergenerational research relationship that integrates anthropological pedagogy. We suggest their initial resistance to this work is the result of age segregation and internalized sociocultural narratives of ageism, as well as misguided expectations of the frustrating, messy, challenging reality of undertaking anthropological research using participant observation. Consistent narrative themes of unprecedented gratitude, personal growth, and learning about ageism emerged from student field notes, class discussions, and written reflections. This program evaluation presents novel insights for future integration of intergenerational programming and gerontological knowledge into anthropological pedagogy.

